# Management of Nonpregnant Women with Elevated Human Chorionic Gonadotropin

**DOI:** 10.1155/2013/580709

**Published:** 2013-10-23

**Authors:** Bernd C. Schmid, Aimee Reilly, Martin K. Oehler

**Affiliations:** ^1^Department of Gynaecological Oncology, Royal Adelaide Hospital, Adelaide, SA 5000, Australia; ^2^Women's and Children's Hospital, North Adelaide, SA 5006, Australia

## Abstract

Human chorionic gonadotropin (hCG) is useful in evaluating and monitoring early pregnancy as well as trophoblastic disease. Here we describe the management of women with elevated serum human chorionic gonadotropin in a case of a 51-year-old female who was unsuccessfully treated for ectopic pregnancy. She was subsequently diagnosed with pituitary hCG production, which should be considered as differential diagnosis before treatment is initiated.

## 1. Short Communication

A 51-year-old parous woman presented with a history of intermittent pelvic cramps and vaginal spotting after two years of amenorrhoea following insertion of an etonogestrel implant for contraception. She was found to have an elevated serum human chorionic gonadotropin (total *β* hCG (hCG + hCG*β*)) measured via a quantitative electrochemiluminescence immunoassay “ECLIA” recognizing the holo-hormone, “nicked” forms of hCG, the *β*-core fragment, and the free *β*-subunit (Elecsys free *β*-hCG, Roche Diagnostics, Germany). Several measurements were performed and total *β* hCG levels of 16.2 to 32.8 IU/L were detected. To exclude a missed abortion, dilatation and curettage were done but the histology excluded any pregnancy products showing secretory endometrium and a benign endometrial polyp. Ectopic pregnancy or gestational trophoblastic disease (GTD) was not identified by subsequent CT scans [1]. To exclude phantom or false positive hCG results caused by unspecific binding of heterophilic serum antibodies, urine hCG was measured and found to be positive [2]. Furthermore heterophilic blocking tube (Scantibodies Laboratory, Inc., USA) pretreatment of the serum samples was performed, and the results were the same as in the original assay [3].

As the clinical picture was consistent with an unidentified ectopic pregnancy, the patient was commenced on methotrexate 50 mg/m^2^ intramuscularly [4]. However, the serum total *β* hCG remained elevated. 

As pregnancy, GTD, and ovarian neoplasia had been excluded, another differential diagnosis was pituitary hCG production [5]. The patient's hormone status was therefore assessed and a follicular stimulating hormone (FSH) of 60.2 U/L (reference intervals: follicular phase 3.5–12.5 U/L, luteal phase 1.5–8.0 U/L, postmenopausal 25–135 U/L) showed postmenopausal levels [6]. To suppress pituitary hCG production the patient was placed on a combined oestrogen-progesterone hormone replacement therapy and after two weeks the serum hCG levels were found to be normal, measuring <2.0 IU/L on serial testing. 

## 2. Discussion

Human chorionic gonadotropin (hCG) is a heterodimeric glycoprotein hormone composed of an *α*-chain (hCG*α*) and a *β*-chain (hCG*β*). hCG*α* is essentially identical to that of luteinizing hormone (LH), follicle-stimulating hormone (FSH), and thyroid-stimulating hormone (TSH), whereas the *β*-chains have greatly differing structures and are responsible for the respective specific hormonal functions and immunological specificity [7]. In pregnancy, intact hCG is the most common form and free hCG*β* accounts for less than 1% of total *β* hCG (hCG + hCG*β*) in serum [8]. Apart from pregnancy, there are other gynaecological conditions that can cause an elevated serum hCG. Gestational trophoblastic diseases such as hydatidiform moles, gestational trophoblastic neoplasias, and choriocarcinomas are diseases where hCG is elevated and the hCG ratio changes in favour of free hCG*β* [9]. Other gynaecological malignancies with elevated hCG include placental site trophoblastic tumours and ovarian germ cell tumors [10].

False-positive results mimicking disease can be related to the assays used to detect hCG. In some tests cross-reactions with other serum glycoproteins results in false-positive hCG levels. Furthermore heterophilic serum antibodies (e.g., anti-mouse antibodies) can bind nonspecifically to antibodies used in the hCG assays, therefore, resulting in false-positive levels [11]. Conversely, a false-negative result may be the result of a hook effect when very high levels of hCG*β* supersaturate the antibodies in the assay and result in false low levels [12].

In gestational trophoblastic neoplasia, aberrant glycosylation and overexpression of the hCG*β* result in hyperglycosylated hCG. Hyperglycosylated hCG shares the same polypeptide structure as hCG but with larger N- and O-linked oligosaccharides. It promotes growth of cytotrophoblast cells and placental implantation in pregnancy as well as invasion of the trophoblast in gestational trophoblastic neoplasia [13, 14].

Although gynaecological malignancies are a common reason for elevated serum hCG in nonpregnant women, it has also been described as paraneoplastic syndrome in other nongynaecological malignancies including cancers of the bladder, kidney, prostate, GI-tract, breast, and lung [10].

Another potential origin of hCG production which is sometimes overlooked due to the common focus on ectopic pregnancy or an underlying malignancy is the pituitary gland in peri- or postmenopausal women [15]. The exact mechanism of hCG production in the gonadotropic cells of peri- or postmenopausal women or after bilateral oophorectomy is unknown. The most likely explanation is the reduction of ovarian steroid hormone synthesis that releases the negative feedback control of gonadotropin-releasing hormone (GnRH). Under this overstimulation, the pituitary may secrete hCG [5].

**Figure 1:**
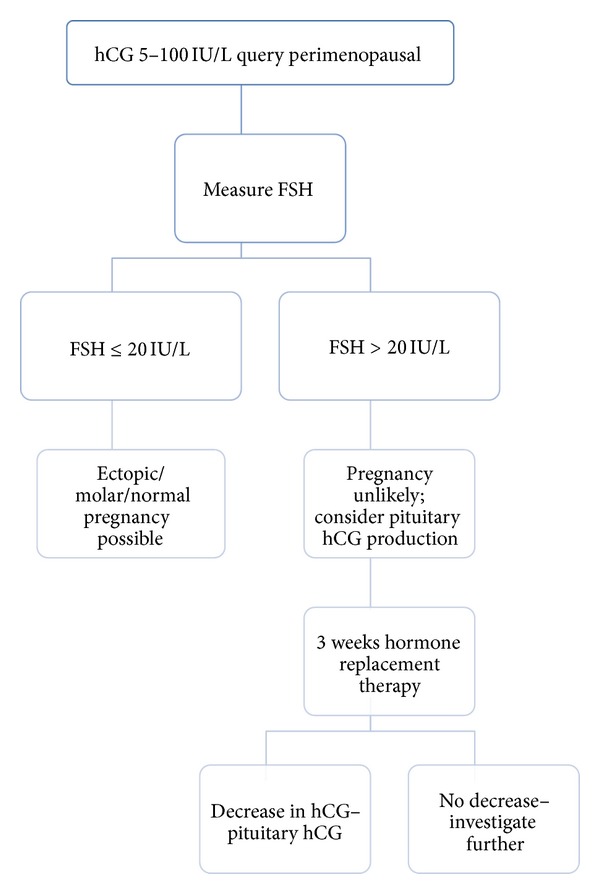
Algorithm for interpreting low serum hCG results in perimenopausal women to exclude pituitary hCG production.

The presented case shows that there is often an incorrect assumption in the medical community that an elevated serum hCG implies that a patient is pregnant or has a trophoblastic disease. Ultimately this resulted in our case in unnecessary and potentially harmful therapy, which could have been easily avoided. Low levels of HCG can be a normal physiological phenomenon in peri- and postmenopausal women. Pituitary hCG is more commonly detected in women greater than 55 years of age but can be detected in women as young as 41 years. Pituitary hCG therefore needs to be excluded in peri- or postmenopausal women (Figure 1).
